# Prognostic significance of crown-like structures to trastuzumab response in patients with primary invasive HER2 + breast carcinoma

**DOI:** 10.1038/s41598-022-11696-6

**Published:** 2022-05-24

**Authors:** Charles N. Birts, Constantinos Savva, Stéphanie A. Laversin, Alicia Lefas, Jamie Krishnan, Aron Schapira, Margaret Ashton-Key, Max Crispin, Peter W. M. Johnson, Jeremy P. Blaydes, Ellen Copson, Ramsey I. Cutress, Stephen A. Beers

**Affiliations:** 1 Antibody and Vaccine Group, Centre for Cancer Immunology, School of Cancer Sciences, Faculty of Medicine, University of Southampton, Southampton, SO16 6YD UK; 2 School of Biological Sciences, Faculty of Environmental and Life Sciences, University of Southampton, Southampton, SO17 1BJ UK; 3 Institute for Life Sciences, University of Southampton, Southampton, SO17 1BJ UK; 4 CRUK Southampton Centre, School of Cancer Sciences, Faculty of Medicine, University of Southampton, Southampton, SO16 6YD UK; 5 Cellular Pathology, University Hospitals Southampton NHS Foundation Trust, Southampton, SO16 6YD UK; 6 Southampton Experimental Cancer Medicine Centre, School of Cancer Sciences, Faculty of Medicine, University of Southampton, Southampton, SO16 6YD UK; 7 NIHR Southampton Biomedical Research Centre, University of Southampton and University Hospital Southampton NHS Foundation Trust, Southampton, SO16 6YD UK

**Keywords:** Breast cancer, Tumour immunology

## Abstract

Obesity can initiate, promote, and maintain systemic inflammation via metabolic reprogramming of macrophages that encircle adipocytes, termed crown-like structures (CLS). In breast cancer the presence of CLS has been correlated to high body mass index (BMI), larger mammary adipocyte size and postmenopausal status. However, the prognostic significance of CLS in HER2 + breast cancer is still unknown. We investigated the prognostic significance of CLS in a cohort of 69 trastuzumab-naïve and 117 adjuvant trastuzumab-treated patients with primary HER2 + breast cancer. Immunohistochemistry of tumour blocks was performed for CLS and correlated to clinical outcomes. CLS were more commonly found at the adipose-tumour border (B-CLS) (64.8% of patients). The presence of multiple B-CLS was associated with reduced time to metastatic disease (TMD) in trastuzumab treated patients with BMI ≥ 25 kg/m^2^ but not those with BMI < 25 kg/m^2^. Phenotypic analysis showed the presence of CD32B + B-CLS was strongly correlated to BMI ≥ 25 kg/m^2^ and reduced TMD in trastuzumab treated patients. Multivariable analysis suggested that CD32B + B-CLS positive tumours are associated with shorter TMD in trastuzumab-treated patients (HR 4.2 [95%CI, (1.01–17.4). This study indicates adipose-tumour border crown-like structures that are CD32B + potentially represent a biomarker for improved personalisation of treatment in HER2-overexpressed breast cancer patients.

## Introduction

Adipose tissue is an important component of the healthy human breast and yet high body mass index (BMI) is associated with increased risk of developing breast cancer in post-menopausal women and with worse survival in all age groups compared to patients with healthy body weight^1^. There is consequently significant interest in understanding the dynamic endocrine and immunological activity of the breast and how high BMI impacts these systems and ultimately influences pathology. Adipose tissue is involved not only in the biosynthesis of important molecular mediators but is also linked to the metastatic potential of breast tumours^2^. It constitutes a dynamic endocrine and immunologically active organ that regulates energy homeostasis, as well as having important immunomodulatory properties^3^. White adipose tissue has evolved as a slowly mobilised energy store in the form of lipids^4^. During weight gain, adipocytes undergo structural changes resulting in the pathological growth of the adipose tissue that leads to adipocyte hypertrophy and adipocyte death^5,6^. The released intracellular contents of the dead adipocytes such as free fatty acids, cytokines and damage-associated molecular patterns, stimulate the recruitment and proliferation of metabolically activated macrophages, that surround and phagocytose the released cellular material, forming so-called crown-like structures (CLS)^7,8^.

Crown-like structures induce a proinflammatory environment and constitute a biomarker of white adipose tissue inflammation and correlate with metabolic dysfunction^7,8^. Inflammation can occur concurrently in both breast and abdominal subcutaneous white adipose tissue as shown in women who underwent bilateral mastectomy and immediate autologous ‘flap’ reconstruction suggesting that CLS are a marker of low-grade systemic inflammation^9^. The presence of CLS is associated with raised levels of serum high sensitivity C-reactive protein (hsCRP), IL-6 and leptin indicating their pro-inflammatory nature^10^. In addition, CLS in obese adipose tissue have a mixed phenotype that is characterised by the concurrent expression of surface markers that describe M1- such as CD11c, and M2-like macrophages such as CD206 or CD163^11^.

In patients with primary breast cancer, CLS found at any location (Any-CLS; distant adipose tissue, within the adipose-tumour border or intratumoural) are present in 36–50% of patients and have been associated with clinical and body composition parameters^10,12^. Specifically, the presence of Any-CLS has been correlated to high BMI, larger mammary adipocyte size and postmenopausal status^10^. Although there is no clear evidence of association between the presence of CLS identified using the pan-macrophage marker CD68 and clinicopathological features^10,12,13^, the presence of Any-CLS is correlated to impaired relapsed free survival and overall survival in women with early breast cancer^10,13,14^. Moreover, CLS identified using the tumour associated macrophage marker CD163 have been correlated to triple negative and HER2 + breast cancer subtypes^13^.

Between 10–25% of patients with early breast cancer show overexpression of the HER2 protein which has been associated with a high risk of relapse in localised disease and poor overall survival in metastatic disease^15–17^. While the introduction of anti-HER2 monoclonal antibodies such as trastuzumab has significantly improved clinical outcomes in both early and metastatic breast cancer^18,19^, resistance to trastuzumab treatment, both primary and acquired, remains a significant clinical challenge^20^. Multiple potential mechanisms of action by which trastuzumab targets HER2 + breast cancers have been proposed^21^. These include its binding to the juxta-membrane portion of the extracellular domain of HER2 receptor^21^ thus preventing the cleavage of the HER2 receptor and the formation of a signalling remnant p95. This consequently reduces the phosphorylation of p95 and blocks signal transduction^21^. Trastuzumab may also inhibit the HER2 signalling pathway by physically blocking either homodimerization or heterodimerization^20^. A third mechanism of action is the recruitment via the antibody Fc of innate immune effectors, leading to tumour-cell killing^21,22^. Additional mechanisms such as HER2 receptor degradation via endocytosis have been proposed.

Supporting a role for antibody effector function in the efficacy of trastuzumab, mice lacking the inhibitory CD32B (FcγRIIB) demonstrated enhanced trastuzumab-dependent cell-mediated cytotoxicity whereas mice deficient in activating FcγR were unable to inhibit tumour growth in mouse xenograft breast tumour model^23^. In mouse xenograft tumour models the increased infiltration of macrophages in the tumour was associated with improved trastuzumab efficacy whereas depletion of macrophages led to reduced antitumor activity^22^. In addition, increased expression of human CD16a (FcγRIII) or mouse FcγRIV by macrophages activated with interferon-γ, induced trastuzumab-mediated antibody-dependent cellular phagocytosis of tumour cells^22^. A recent study that included 40 patients with HER2 + metastatic breast cancer who were treated with trastuzumab, showed that increased numbers of iNOS + M1-like macrophages and CD8 + T-cells within the tumour was associated with improved survival, indicating that responses to trastuzumab can be affected by the immunological status of the tumour^24^.

Adiposity has been shown to affect the local immune environment of solid tumours in both pre-clinical and clinical studies where several studies suggest that obesity potentially enhances responses to immune checkpoint inhibitors^25,26^. Nevertheless, the evidence for the role of adiposity in therapeutic response in HER2 + breast cancer is conflicting which may be attributed to the heterogeneity of breast tumours. In the metastatic setting, a multicentre retrospective cohort study showed that BMI was not associated with clinical outcomes in patients with metastatic HER2 + breast cancer^27^. In contrast, Krasniqi E et al., showed that BMI ≥ 30 kg/m^2^ was correlated with poor overall survival in patients with HER2 + metastatic breast cancer treated with pertuzumab and/or trastuzumab emtansine^28^.

In early breast cancer, Di Cosimo et al. demonstrated that patients with high BMI and HER2 + oestrogen receptor + (ER +) early primary breast cancer achieved lower complete pathological responses compared to patients with healthy or underweight BMI, after receiving neoadjuvant treatment with anti-HER2 + agents^29^. Furthermore, Mazzarella et al. reported that BMI ≥ 30 kg/m^2^ was correlated with shorter overall survival and increased incidence of distant metastases in HER2 + ER− breast cancer whereas the outcome in HER2 + ER + tumours did not significantly differ between the different BMI groups^30^. Together, these findings indicate that adiposity and hormonal profile may explain the differential responses to anti-HER2 agents in patients with HER2 + primary breast cancer.

The spatial distribution of immune and tumour cells has been correlated to clinical outcomes in solid tumours. In patients with adenocarcinoma of the lung, immune geospatial variability was associated with clinical outcomes^31^. Similarly, in patients with triple negative breast cancer, multiplexed imaging of immune cells and tumour markers revealed that tumour expression and immune composition were interrelated and associated with overall survival^32^. The geospatial distribution of immune cell composition was also investigated in patients with HER2 + breast cancer treated with neoadjuvant HER2-targeted therapy^33^. This study demonstrated the importance of spatial proteomic profiling of tumour-immune microenvironment in capturing regional tumour heterogeneity and treatment-associated changes. Currently, there is no clear evidence on the effect of the spatial distribution of CLS on therapeutic responses in patients with HER2 + breast cancer.

The prognostic significance of CLS and consequently of white adipose tissue inflammation in patients with HER2 + primary breast cancer is largely unknown. In the current exploratory study, we investigated the phenotype and spatial distribution of CD68 + CLS in a small cohort of 188 HER2 + breast cancer patients and correlate these phenotypes to clinical outcomes. We showed that the presence of CD32B + CLS at the adipose-tumour border are associated with shorter time to metastatic disease in patients with HER2-overexpressed breast tumours who were treated with adjuvant trastuzumab. Thus, CD32B + adipose-tumour border CLS could potentially be used as a biomarker to optimise patient stratification and personalisation of treatment in HER2-overexpressed breast cancer patients.

## Materials and methods

### Study population

This study was conducted in a retrospective series of 188 patients with HER2 + invasive breast carcinomas who were diagnosed with breast cancer between 1982 to 2012 at the University Hospital Southampton NHS Foundation Trust, UK. Inclusion criteria were histological confirmation from clinical pathology reports of HER2 positivity (immunohistochemistry score 3 + or immunohistochemistry score 2 + with positive in-situ hybridisation), and surgical excision of the tumour at University Hospital Southampton NHS Foundation Trust. This cohort includes a HER2 + trastuzumab naïve group of 69 unselected primary operable (stage I-III) HER2 + invasive breast carcinomas presenting between 1982 and 2004 and a HER2 + adjuvant trastuzumab group consisting of 117 primary operable HER2 + breast tumours from patients diagnosed between 2005 and 2012. All patients were treated in a single institution with surgery (mastectomy or wide local excision and axillary surgery), followed by adjuvant chemotherapy and/or radiotherapy as per standard surgical and oncological protocols. Adjuvant trastuzumab was given intravenously with a loading dose of 8 mg/kg and maintenance dose of 6 mg/kg every 3 weeks. The planned duration of adjuvant trastuzumab was 12 months. Clinicopathological parameters for both case series are summarised in Supplementary Table 1. Patients with BMI < 18.5 kg/m^2^ or de novo metastatic disease or patients who were treated with neoadjuvant chemotherapy were excluded from the study.

Survival data, including time to metastatic disease and development of loco-regional and distant metastases were extracted from clinical records. Time to metastatic disease was defined as the number of months from first operation to the occurrence of distant recurrence. Breast cancer distant disease-free survival was censored if the patient was still alive and progression-free at the time of analysis, lost to follow-up, or died by other causes.

Ethical approval was obtained from South Central – Hampshire B Research Ethics Committee (10/H0504/73). Tumour Marker Prognostic Studies (REMARK) criteria informed the reporting of this study.

### Immunohistochemistry

Immunohistochemistry for CD16, CD32B and CD68 were performed on 4 µm sections of formalin-fixed paraffin-embedded breast tumours using BOND MAX Fully Automated Research Stainer, Leica Microsystems, U.K. using BOND reagents according to the manufacturer’s instructions as previously reported^34^. Sections were deparaffinized, pre-treated for heat-induced Ag retrieval (BOND ER1/ER2 protocol) and incubated with hydrogen peroxide followed by primary antibody. Mouse or rabbit Abs were subsequently bound to the Post Primary IgG linker reagent or the Poly-HRP IgG reagent, respectively, before incubation with 3,3′-diaminobenzidine (DAB). The sections were subsequently incubated with the other primary antibody, which was then bound to either the Post Primary IgG linker reagent or the Poly-HRP IgG reagent. The substrate chromogen Fast Red was applied and sections counterstained using haematoxylin and mounted in CV Ultra mounting media (Leica Microsystems).

Antibodies used were anti-CD16 (FcγRIII) (Abcam,2H7) at 1:100 dilution anti-CD32B (FcγRIIB) (Abcam, EP888Y) at 1:3000 dilution and anti-CD68 (DAKO, PG-M1) at 1:250 dilution. Negative control by substitution of the primary antibody with IgG-matched serum was included in each run. Formalin-fixed and paraffin-embedded CHO-k1 human CD16 and human CD32B transfected cells and human tonsil were used as positive controls.

### CLS quantification method

CLS were defined as a structure consisting of an adipocyte encircled by CD68 + macrophages by ≥ 50%. The presence and number of CD68 + , CD16 + CD68 + and CD32B + CD68 + CLS were evaluated manually in full face sections using a Nikon Eclipse 80i microscope (Nikon, Tokyo, Japan) by two independent scorers (CB and AL) who were blinded to patients’ clinicopathological data and outcomes. CLS were defined as CLS within distant adipose tissue (D-CLS) if the CLS were located > 1 mm away from the adipose tissue-tumour interface into the adipose tissue, CLS within the adipose-tumour border (B-CLS) if the CLS were located ≤ 1 mm away from the adipose tissue-tumour interface into the adipose tissue and intratumoural CLS (T-CLS) if the CLS were located in the tumour/stromal tissue and not in the adipose tissue. Any CLS was determined if a patient had a CLS in at least one compartment. The cut-off of CLS ≤ 1 per full-face section was used to differentiate low from high CLS.

### Statistical analysis

Statistical analysis was performed using STATA (StataCorp, version 16, Texas, USA). All available data were analysed to increase power and minimise the introduction of selection bias. Baseline characteristics of both cohorts by detection of CLS at the adipose-tumour border were summarised by mean and standard deviation for continuous variables and frequencies and percentages for categorical variables. Where appropriate, Pearson’s Chi-square, Fisher’s exact, Student’s t and ANOVA one-way tests were used. For multiple comparisons, p values were adjusted according to Benjamini and Hochberg multiple p value adjustment method^35^. Cumulative survival probabilities were estimated using the Kaplan–Meier method, and differences between survival rates were tested for significance using the log-rank test. Univariate analysis was performed and where variance was significant, this was carried forward to multivariable analysis. Multivariable analysis for time to metastatic disease against covariates CD32B + B-CLS and white cell count was performed using the Cox proportional hazard model. Potential confounders were selected for inclusion in multivariable model based on known associations in the literature. Hazard ratios (HR) and 95% confidence intervals (95% CI) were estimated for each variable. All tests were two-sided with a 95% CI and a p value < 0.05 considered significant.

### Ethical approval

All procedures performed in studies involving human participants were in accordance with the ethical standards of the institutional and/or national research committee and with the 1964 Helsinki declaration and its later amendments or comparable ethical standards. Ethical approval was obtained from South Central – Hampshire B Research Ethics Committee (10/H0504/73). All data were anonymised, and all the personal identifiers were removed. Informed consent was obtained for research use of tissue surplus to diagnostic requirements as part of the routine surgical consent form.

## Results

### Patient characteristics

The whole cohort in this exploratory study consisted of 188 patients with HER2 + primary breast cancer. The baseline characteristics of the whole cohort are described in Supplementary Table 1. Missing values for clinical parameters are shown in Supplementary Table 2. A total of 176 tumours were suitable for CLS quantification and analyses. The median follow-up was 36.6 months (range, 2.6–299.1). 117 patients were treated with adjuvant trastuzumab and 69 patients, who were diagnosed prior to 2005 were trastuzumab naïve. The number of patients with a distant recurrence was 11 in 108 trastuzumab treated patients and 29 in 65 trastuzumab-naïve patients (Supplementary Tables 3 and 4 respectively).

### Frequency and spatial distribution of CLS by BMI

**Figure 1:**
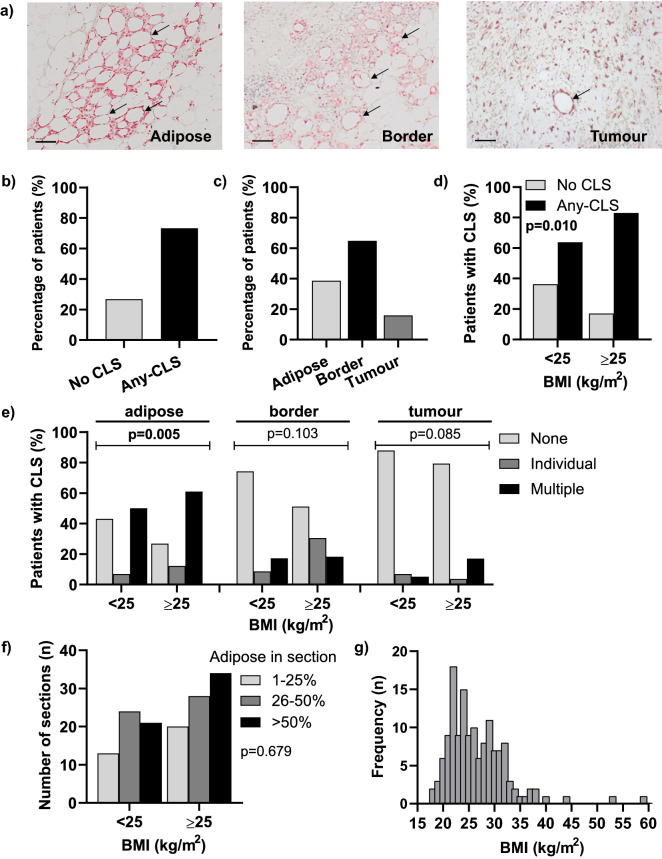
Frequency and spatial distribution of CLS. (**a**) Representative images showing the spatial distribution of CLS either in the adipose, at the adipose-tumour border, or within the tumour. Arrows indicate examples of CLS at each location. Scale bar = 100 μm; Refine red, CD68; (**b**) Percentage of patient tissue sections in cohort that contained at least 1 CLS in any location; (**c**) Spatial distribution of CLS in patients with at least 1 CLS; (**d**) Presence of at least 1 CLS in any location stratified by BMI; (**e**) CLS quantification stratified by frequency, spatial distribution, and BMI; (**f**) Proportion of adipose tissue in each tissue section stratified by BMI; (**g**) Distribution of patient BMI across the whole cohort is representative of the general population. P values were calculated with Pearson’s Chi-square or Fisher exact test if one or more of cells had an expected frequency of 5 or less.

We evaluated the presence of CLS in the adipose (D-CLS), border (B-CLS) and tumour (T-CLS) as shown in representative images in Fig. 1a. In the whole cohort, at least one CLS per full-face section (Any-CLS) was detected either within the distant adipose tissue, the adipose-tissue border, or the tumour in 129 (73.3%) tumour sections (Fig. 1b). CLS were more commonly found in the adipose-tumour border (64.8%) rather than in the distant adipose tissue (38.6%) or intratumorally (15.9%) (Fig. 1c). The presence of Any-CLS was significantly associated with BMI ≥ 25 kg/m^2^ (p = 0.01) (Fig. 1d). We then assessed the spatial distribution of CLS and found that detection of D-CLS was associated with BMI ≥ 25 kg/m^2^ (p = 0.005) (Fig. 1e). Although there was a trend for positive correlation between individual or multiple B-CLS and BMI ≥ 25 kg/m^2^ this was not statistically significant (p = 0.103). A similar trend was observed for T-CLS which was also non-significant (p = 0.085). To control for bias in scoring, we also evaluated the correlation between the proportion of adipose tissue per tissue section and BMI, which showed no evidence of association (Fig. 1f). Finally, we assessed the BMI distribution across the whole cohort (Fig. 1g) which demonstrated that the BMI distribution of this cohort is representative of the general population^36^.

### Association between CLS phenotype and BMI

**Figure 2:**
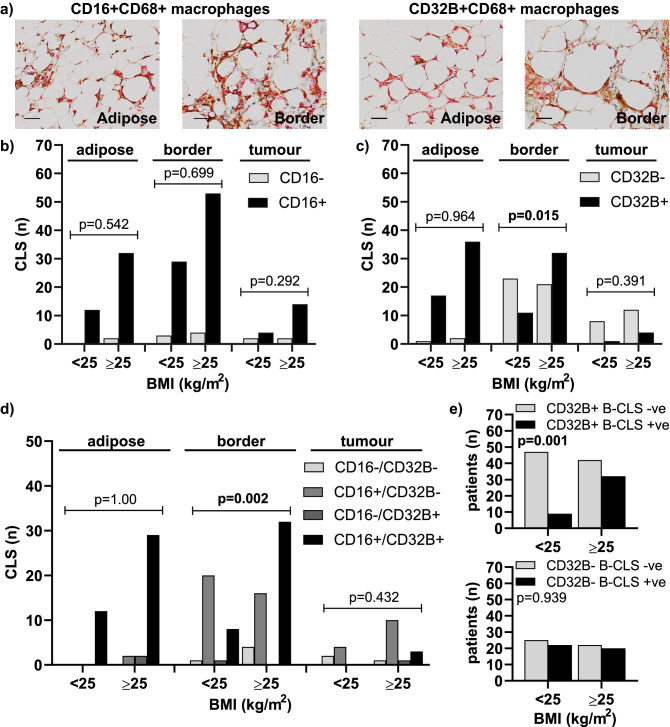
Macrophage phenotype within adipose tissue and at the adipose-tumour border stratified by BMI. (**a**) Representative images of CD16 + CD68 + and CD32B + CD68 + CLS within the adipose tissue and at the adipose-tumour border. Scale bar = 50 μm; Refine red, CD68; DAB, CD16 or CD32B; (**b**) Spatial distribution of CD16 + CD68 + and (**c**) CD32B + CD68 + CLS; (**d**) Co-expression analysis of CD16 and CD32B in CLS stratified by spatial distribution and BMI; (**e**) Association between CD32B + B-CLS (top) and CD32B- B-CLS (bottom) with BMI within the whole cohort. P values were calculated with Pearson’s Chi-square or Fisher exact test if one or more of cells had an expected frequency of 5 or less.

CLS are characterised by a mixed macrophage phenotype with CD16 expression being predominantly expressed^37^. For this reason, we assessed the expression of CD16 (FcγRIII), an activating Fcγ receptor, and CD32B (FcγRIIB), the inhibitory Fcγ receptor, by CLS macrophages in the different tissue compartments. Figure 2a shows representative images of CD16 + and CD32B + CLS from the distant adipose tissue and adipose-tumour border. Evaluation of the phenotype of CLS showed no evidence of association between the expression of CD16 in CLS and BMI in the distant adipose tissue, the adipose-tumour border or in the tumour when CLS were present in those locations (Fig. 2b). Although the expression of CD32B in D-CLS and T-CLS were not correlated with BMI ≥ 25 kg/m^2^, there was a positive association between CD32B expression in B-CLS and BMI ≥ 25 kg/m^2^ (p = 0.015) (Fig. 2c). Subsequently, we assessed the co-expression of CD16 and CD32B in CLS in distant adipose tissue, adipose-tumour border, and tumour. This demonstrated that double positive expression of CD16 and CD32B in B-CLS was positively associated with BMI ≥ 25 kg/m^2^ (p = 0.002) (Fig. 2d). Whilst we had found no association between the presence of B-CLS and BMI in the whole cohort (Fig. 1e), consistent with this BMI-associated change in the CD32B status of the B-CLS, there was a positive association between the presence of CD32B positive B-CLS and increased BMI ≥ 25 kg/m^2^ (p = 0.001) (Fig. 2e).

### Prognostic significance of CLS on time to metastatic disease

**Figure 3:**
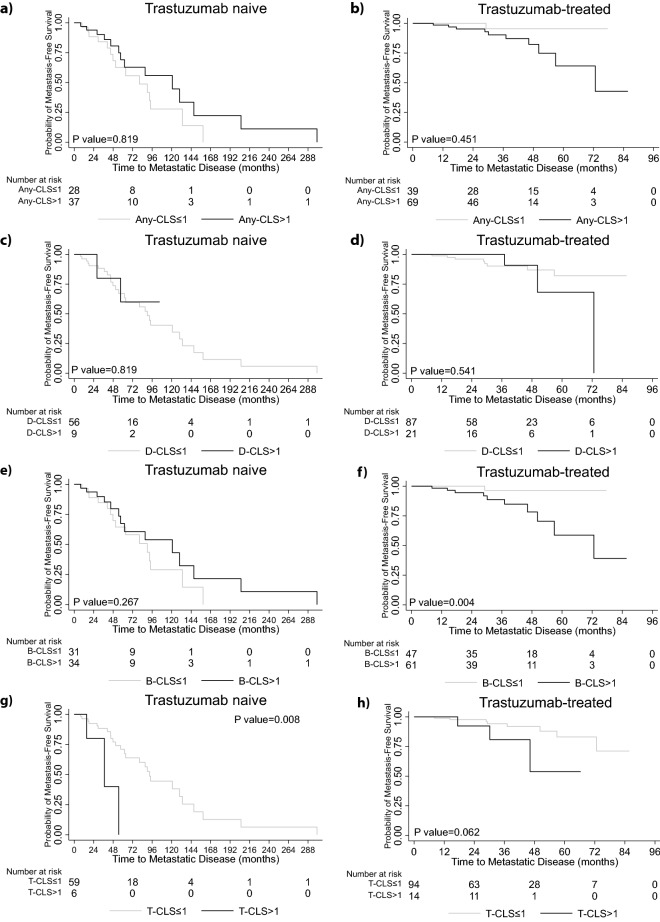
Kaplan–Meier curves showing time to metastatic disease in trastuzumab naïve and trastuzumab-treated patients. (**a,b**) Any-CLS ≤ 1 versus Any-CLS > 1; (**c,d**) D-CLS ≤ 1 versus D-CLS > 1; (**e,f**) B-CLS ≤ 1 versus B-CLS > 1; (**g,h**) T-CLS ≤ 1 versus T-CLS > 1.

Next, we investigated the potential prognostic significance of CLS stratified by trastuzumab treatment status. Any-CLS and D-CLS were not associated with time to metastatic disease, neither in the trastuzumab naïve, nor in the trastuzumab treated patients (Fig. 3a–d). When stratified by B-CLS, B-CLS were not associated with clinical outcome in the trastuzumab-naïve patients. Conversely in the trastuzumab treated patients there was a significant reduction in time to metastatic disease in those with more than one adipose-tumour border CLS (B-CLS > 1) (p = 0.004) (Fig. 3e,f).

In the trastuzumab-naïve arm, the estimated 2-year and 5-year time to metastatic disease in B-CLS ≤ 1 patients was 88.6% [95%CI, (68.6–96.2)] and 64.9% [95%CI, (41.4–80.9)], respectively. In the same group, patients with B-CLS > 1 had 93.5% [95%CI, (76.4–98.3)] and 67.6% [95CI%, (42.4–83.7)] 2-year and 5-year time to metastatic disease (Fig. 3e). In the trastuzumab-treated arm, the estimated 2-year and 5-year time to metastatic disease for the B-CLS ≤ 1 patients were 100% and 96.7% [95%CI, (78.6–99.5)], respectively. In the same treatment group, the 2-year and 5-year time to metastatic disease for the B-CLS > 1 patients was 94.3% [95%CI, (83.2–98.1)] and 61.4% [95%CI (33.1–80.6)], respectively (Fig. 3f).

T-CLS > 1 was significantly associated with shorter time to metastatic disease (p = 0.008) in the trastuzumab naïve patients whereas there was a non-significant trend for this association in those that received trastuzumab treatment (p = 0.062) (Fig. 3g,h). However, these data are based on a relatively small number of patients with T-CLS present, n = 6 and 14 for T-CLS > 1 in trastuzumab-naïve and trastuzumab-treated patients respectively. Overall though, these findings indicate that the spatial distribution of CLS may determine their prognostic impact.

### Correlations between B-CLS and clinical parameters

As shown above, CLS were frequently detected at the adipose-tumour border compared to other tissue compartments (Fig. 1c) and B-CLS were correlated with clinical outcomes in the trastuzumab treated patients (Fig. 3f). In addition, CD32B + expression in B-CLS was correlated to BMI ≥ 25 kg/m^2^ (Fig. 2e). For this reason, the subsequent analysis focused on B-CLS.

Patient characteristics stratified by B-CLS status in trastuzumab-naive and trastuzumab-treated cohort arms are shown in Supplementary Tables 5 and 6. In the trastuzumab-naive arm, B-CLS ≤ 1 and B-CLS > 1 were present in 31 (47%) and 35 (53%), respectively. In the trastuzumab-treated arm, B-CLS ≤ 1 were identified in 47 (43.5%) of the tumours whereas B-CLS > 1 were found in 61 (56.5%). There was no evidence of association between the presence of B-CLS and clinicopathological characteristics in either cohort arms (adjusted p > 0.05). The mean duration of adjuvant trastuzumab was 10.8 and 10.2 months in the B-CLS ≤ 1 group and B-CLS > 1 group, respectively. The presence of multiple B-CLS was associated with a higher peripheral blood white cell count both in the trastuzumab-naïve (p = 0.04) and the trastuzumab-treated patients (p = 0.06). However, this became non-significant after controlling for multiple comparisons.

### Prognostic significance of B-CLS by BMI

**Figure 4:**
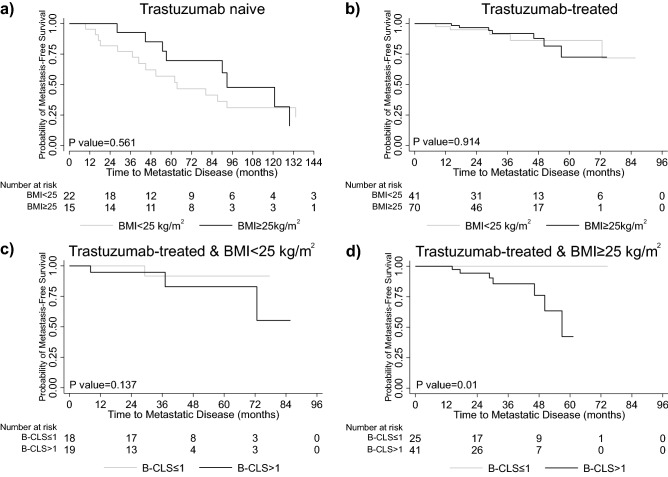
Kaplan–Meier curves showing time to metastatic disease in trastuzumab naïve and trastuzumab-treated patients. Comparison of patients with (**a,b**) BMI < 25 kg/m^2^ versus BMI ≥ 25 kg/m^2^; (**c,d**) Analysis of trastuzumab-treated only patients stratified by BMI comparing B-CLS ≤ 1 versus B-CLS > 1.

In both the trastuzumab-naïve and the trastuzumab-treated cohort arms, BMI was not associated with time to metastatic disease (Fig. 4a,b). Stratified analysis of trastuzumab-treated patients by BMI showed that BMI may have an impact on the prognostic effect of B-CLS. Specifically, there was no statistically significant difference between B-CLS status and time to metastatic disease in patients with BMI < 25 kg/m^2^ (p = 0.137) (Fig. 4c). However, in the BMI ≥ 25 kg/m^2^ group, patients with B-CLS > 1 had shorter time to metastatic disease compared to patients with B-CLS ≤ 1 (p = 0.01) (Fig. 4d).

### Prognostic significance of B-CLS in trastuzumab-treated patients by Hormone Receptor status

The association of BMI and B-CLS with clinical outcomes in the trastuzumab-treated patients was stratified by hormone receptor status. As demonstrated in Supplementary Fig. 1a,b, BMI ≥ 25 kg/m^2^ was not associated with time to metastatic disease neither in the HER2 + ER− nor in HER2 + ER + cohorts. Although there was weak evidence of possible association between the presence of B-CLS > 1 and clinical outcomes in the HER2 + ER− cohort (p = 0.099) (Supplementary Fig. 1c), there was evidence of association between the presence of B-CLS > 1 and reduced time to metastatic disease in the HER2 + ER + cohort (p = 0.019) (Supplementary Fig. 1d). In patients with BMI ≥ 25 kg/m^2^, the presence of B-CLS > 1 in HER2 + ER− breast tumours was associated with shorter time to metastatic disease (p = 0.031) (Supplementary Fig. 1e) whereas there was a trend for a possible association in the HER2 + ER + patients (p = 0.097) (Supplementary Fig. 1f). These findings suggest that the prognostic association of B-CLS on clinical outcomes to trastuzumab treatment may be influenced by HER2/ER co-expression profile and BMI.

### Prognostic significance of CD32B + B-CLS by BMI

**Figure 5:**
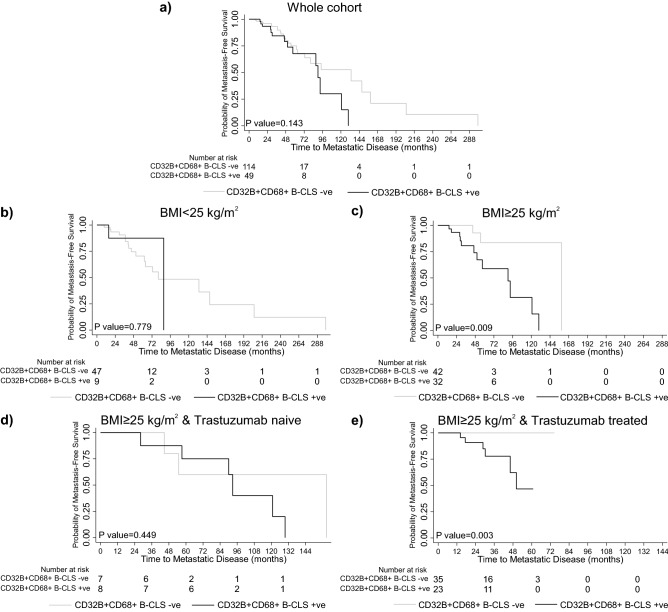
Kaplan Meier curves showing time to metastatic disease in patients with CD32B + B-CLS. (**a**) All patients in cohort. (**b,c**) Patients stratified by BMI. (**d,e**) Patients with BMI ≥ 25 kg/m^2^ stratified by trastuzumab treatment status.

As previously shown, the presence of CD32B + B-CLS was correlated with BMI ≥ 25 kg/m^2^, thus we wanted to investigate the potential prognostic significance of the presence of CD32B + B-CLS in the tumours. Survival analysis revealed that the presence of CD32 + B-CLS was not associated with reduced time to metastatic disease across the whole cohort (p = 0.143) (Fig. 5a). However, stratification of the cohort by BMI, revealed a significant association between the presence of CD32B + B-CLS and reduced time to metastatic disease in patients with BMI ≥ 25 kg/m^2^ (p = 0.009) but not with BMI < 25 kg/m^2^ (p = 0.779) (Fig. 5b,c). Patients with BMI ≥ 25 kg/m^2^ were further stratified on trastuzumab treatment status. This showed no evidence of association between the presence of CD32B + B-CLS and time to metastatic disease in trastuzumab naïve patients (p = 0.449) (Fig. 5d). In the trastuzumab-treated group though, there was a significant association between CD32B + B-CLS and shorter time to metastatic disease compared to patients with no CD32B + B-CLS in patients with BMI ≥ 25 kg/m^2^ (p = 0.003) (Fig. 5e). Survival analysis comparing the presence of CD32B− B-CLS with patients with no B-CLS showed no association with BMI or trastuzumab treatment (p > 0.05) (Supplementary Fig. 2). Together these data suggest that the association between CD32B + B-CLS and reduced time to metastatic disease might be driven by B-CLS CD32B positivity rather than just the number of B-CLS alone in high BMI patients.

### CD32B + B-CLS are independent prognostic factors for time to metastatic disease

Multivariable cox regression analysis suggested that the presence of at least one CD32B + B-CLS is an independent prognostic factor for shorter time to metastatic disease in patients with primary HER2 + breast cancer that received adjuvant trastuzumab, HR 4.2 [95%CI, (1.01–17.4)], p = 0.048 (Table 1). The presence of multiple CD32B + B-CLS increased the risk of metastatic disease to 4.9 [95%CI, (1.2–20.6)], p = 0.029 (Supplementary Table 7).

**Table 1:** Univariate and multivariable analysis for time to metastatic disease of the trastuzumab-treated patients comparing the presence of CD32B + B-CLS +ve versus CD32B + B-CLS −ve patients.

Variable	N^a^	Univariate HR (95%CI)	p-value	Multivariable HR (95%CI)	p-value
CD32B + B-CLS +ve versus CD32B + B-CLS −ve ^b^	99	4.6 (1.1–18.3)	**0.038**	4.2 (1.01–17.4)	**0.048**
BMI ≥ 25 versus < 25 kg/m^2^	111	1.1 (0.3–3.5)	0.914		
T3/4 versus T1/2 tumour stage ^c^	116	2.8 (0.8–10.4)	0.123		
N2/3 versus N0/1 nodal stage ^c^	115	3.8 (0.8–18.7)	0.097		
Tumour grade 3 versus grade 1/2 ^d^	116	0.9 (0.2–3.2)	0.841		
HER2 + ER− versus HER2 + ER +	115	1.3 (0.4–4.2)	0.642		
White cell count (≥ 9 versus < 9 10^9^/L)	112	3.8 (1.2–12.6)	**0.028**	2.10 (0.5–8.9)	0.315
Neutrophils (≥ 5.4 versus < 5.4 10^9^/L)	112	2.8 (0.9–8.8)	0.072		
Platelets (≥ 290 versus < 290 10^9^/L)	112	2.7 (0.9–8.3)	0.094		

B-CLS, CLS at the adipose-tumour border; ^a^number of cases for which data was available; ^b^CD32B + B-CLS -ve, patients with CD32B- B-CLS, no B-CLS or any CLS; ^c^as per TNM Classification of Malignant Tumours (8th edition); ^d^as defined by Nottingham grading system; Bold, statistically significant.

## Discussion

Adipose tissue is infiltrated by significant numbers of macrophages via two main mechanisms: either they can differentiate from bone-marrow-derived monocytes that migrate to the adipose tissue by diapedesis from the systemic circulation, or they can transdifferentiate from pre-adipocytes and mesenchymal stem cells within the adipose tissue^38^. Phenotypic differences exist in adipose tissue macrophages between those found in lean and obese adipose tissue. Differing immune phenotypes are driven by a range of activating stimuli such as metabolic ligands including free fatty acids, high-density lipoproteins, and glucose; along with cytokines and pattern recognition receptor ligands^37,39^. In obesity, adipose tissue macrophages are metabolically activated, and their main function is to clear necrotic adipocytes via either phagocytosis or lysosomal activation^37,40^. Free fatty acids released from ruptured adipocytes can initiate the intracellular NF-κB-mediated signalling pathway via the TLR4 receptor, leading to the activation of innate immune cells in the presence of interferon gamma^41,42^. Proinflammatory macrophages are recruited around necrotic or pyroptotic hypertrophic adipocytes forming CLS that secrete proinflammatory mediators such as TNF-α, IL-6, IL-1b, MCP-1, MIF and nitric oxide (NO). Previous studies demonstrated varying degrees of association between the presence of CLS and clinical outcomes in breast cancer^10,13,14^. The study population of these cohorts was predominantly patients with ER + breast cancer. Hence, the prognostic significance of CLS, and consequently of white adipose tissue inflammation, in patients with primary HER2 + breast cancer has not been previously assessed.

The formation of B-CLS are not unique to HER2 + breast cancer and have also been observed in other subtypes including luminal A, luminal B and TNBC^13^. The mechanism of CLS formation has been extensively reviewed elsewhere^7,8^, thus in this exploratory study, we focused our investigation into the phenotype and prognostic role of CLS in 188 patients with HER2 + primary breast cancer. To our knowledge, this is the first study that reports the role of CLS on therapeutic responses in patients with HER2 + breast cancer. CLS were more frequently detected at the adipose-tumour border (Fig. 1c) and were significantly correlated to BMI ≥ 25 (Fig. 1d,e) which is consistent with previous studies^9,10,12,13,43–45^. Adipose tissue macrophages constitute a diverse population that is characterised by a mixed inflammatory phenotype^37^. In high-fat diet mice, obesity was shown to switch the polarisation of the adipose tissue macrophages from an M2- to M1-like phenotype^46^. However, human CLS have a mixed phenotype that is characterised by the concurrent expression of surface markers that describe M1- and M2-like macrophages^11^. CD16 (FcγRIII) is an activating receptor while CD32B (FcγRIIB) is inhibitory, and these tend to be negatively associated whereby in inflammatory M1-like macrophages CD16 is high while CD32B will be low, and visa-versa in M2-like macrophages. Here, we showed that distant adipose-CLS are characterised by being double positive for both CD16 and CD32B, independently of BMI (Fig. 2d). In patients with BMI < 25 kg/m^2^, B-CLS tended to be CD16+/CD32B−. However, in patients with BMI ≥ 25 kg/m^2^, B-CLS were predominantly characterised by a CD16 + CD32B + phenotype (Fig. 2d) suggesting that the higher level of adiposity is potentially driving metabolic dysfunction within CLS at the adipose tumour border. In overweight and obese patients, the presence of CD32B + B-CLS was strongly associated with shorter disease-free survival compared to patients with normal BMI (Fig. 5). Subgroup analysis showed that the presence of CD32B + B-CLS was associated with worse disease-free survival in trastuzumab-treated patients with BMI ≥ 25 kg/m^2^. Multivariable analysis revealed that the presence of CD32B + B-CLS is an independent prognostic factor for metastatic disease (Table 1).

Previous studies have shown varying degrees of association between the presence of CLS and poor clinical outcomes in patients with breast cancer^10,13,14^. This discrepancy in the clinical outcomes in the different cohorts can be attributed to patient and tumour heterogeneity, small sample size and the different quantification methods and outcome assessments. Specifically, these cohorts included patients of any hormonal status and evaluated the presence of CLS within the whole observed adipose tissue area. Geospatial proteomic and transcriptomic profiling of the immune-tumour microenvironment has been associated with clinical outcomes in patients with breast cancer^32,33^. Keren L *et. al.,* showed that the tumour-immune border is a unique site of immune inhibition with altered expression profiles by both tumour and immune cells in patients with triple-negative breast cancer^32^. Hence, the spatial study of immune cell phenotypes and composition as well as their interactions with tumour cells is important in detecting tumour heterogeneity in relation to therapeutic responses. Despite this, to our knowledge no studies have so far directly assessed how the spatial proximity of breast CLS to the adipose/ tumour border may impact prognosis and response to therapy^8^. For this reason, in this study, we set out to investigate the significance of spatial distribution of CLS on clinical outcomes. The spatial distribution of CLS were classified as either being located within the distant adipose tissue (D-CLS); at the adipose-tumour border (B-CLS); or intratumoural CLS (T-CLS) if the CLS were located in the tumour/ stromal tissue and not in the adipose tissue. This investigation has highlighted the potential impact of spatial distribution of CLS within the distant adipose tissue versus the adipose-tumour border revealing clinically important differences compared to these previous studies. Thus, we hypothesise that the presence of CD32B + B-CLS induces, or are at least indicative of, a chronic inflammatory environment that is associated with resistance to anti-HER2 therapy in patients with BMI ≥ 25 kg/m^2^. Due to the multi-modal nature of the mechanism of action of trastuzumab, either by direct cell targeting or engagement with effector cell function^21,47^, the biological mechanism underlying this association between CD32B + B-CLS and trastuzumab resistance is currently unclear and requires further investigation. We can speculate that this may be via a direct effect on the immune system or instead could be a marker of the overall inflammatory state of the tumour microenvironment. Future investigations including the analysis of other immune cell populations such as T-infiltrating lymphocytes, may help to further elucidate the immune mechanism involved.

Epidemiological studies have revealed that the presence of CLS in breast tumours is associated with raised inflammatory markers. Specifically, these studies showed strong evidence of association between high levels of high sensitivity CRP and IL-6 with the presence of CLS in patients with breast cancer^10,44^. The effect of BMI in peripheral inflammation was shown in a cohort of breast cancer patients with healthy BMI where these correlations were either weak or non-significant^43^. These findings indicate that peripheral inflammation may be prominent in patients with high BMI and CLS which in turn may potentially play a critical role in therapeutic responses.

There is also strong evidence of correlation between elevated levels of serum leptin and adiponectin with the presence of CLS^10^. Preclinical studies revealed that leptin activates RAS-dependent MAPK pathway and upregulates the levels of chaperone heat-shock protein 90 (Hsp90) by inducing JAK2/STAT3 activation^48^. These lead to an enhanced HER2 receptor overexpression that reduces sensitivity of breast cancer cells to anti-oestrogen tamoxifen treatment^48^. Leptin has also been implicated in trastuzumab resistance whereby differentiated adipocytes negatively affected trastuzumab-induced growth inhibition of HER2 + BT474 and SKBR3 cell lines, an effect that was mediated via AKT phosphorylation^49^. Inhibition of PI3K suppressed the ability of differentiated adipocytes to promote resistance to trastuzumab^10,49^. Leptin also induces overexpression of HER2 receptor and cell proliferation via the phosphorylation of both epidermal growth factor receptor and Janus-activated kinase 2 (JAK2) in SKBR3 cells^50^.

Together with our data, these findings suggest that a possible underlying mechanism is that adiposity in patients with high BMI induces white adipose tissue inflammation, increasing adipokine expression and metabolic dysfunction. This may be mediated via a paracrine interaction between macrophages, adipocytes and tumour cells leading to the secretion of pro-inflammatory cytokines and adipokines in the breast tissue in obese or overweight patients. This in turn may induce trastuzumab resistance via the activation of MAPK or PI3K signalling pathways. Our observation that this association is driven by CD32B positivity of B-CLS rather than the number of B-CLS alone in high BMI patients may thus potentially act as a novel biomarker allowing for stratification of patients to determine more effective therapeutic strategies. For example, high BMI patients identified to have CD32B + B-CLS are likely to have a reduced response to trastuzumab therapy. Thus, we hypothesise that this subset of patients may benefit from more intensive anti-HER2 therapy such as dual antibody therapy using trastuzumab and pertuzumab in combination. Alternatively, if CD32B + B-CLS are a marker of an immune suppressive tumour environment, then the trastuzumab drug conjugate ado-trastuzumab emtansine (Kadcyla) may be more effective due to its cytotoxic mechanism of action. Conversely, this study highlights how effective trastuzumab treatment is in patients that do not have CD32B + B-CLS, and so these patients could benefit from de-escalation of anti-HER2 therapy thereby minimising potential side-effects. However, it is important to highlight the exploratory nature of this present study and that the results are based on a relatively small number of patients. Thus, further validation studies using additional cohorts of patients with primary HER2 + breast cancer treated with trastuzumab is required to confirm these findings. Also, the median follow-up of 36.6 months in our cohort does limit the results’ interpretation and so future studies should thus aim to include longer follow-up times.

In conclusion, we provide evidence which indicates that the presence of B-CLS correlates with clinical outcomes and therapeutic responses in patients with HER2-overexpressed breast cancer. Also, CD32B positivity of B-CLS may represent a predictive biomarker which could potentially be used to optimise the stratification and personalisation of treatment in HER2-overexpressed breast cancer patients.

## Supplementary Information

Supplementary Information.

## Data Availability

The datasets used and/or analysed during the current study are available from the corresponding author on reasonable request.

## Abbreviations

BMI: Body mass index
CLS: Crown-like structures
D-CLS: CLS within distant adipose tissue
B-CLS: CLS within the adipose-tumour border
T-CLS: Intratumoural CLS
ER: Oestrogen receptor
hsCRP: High sensitivity C-reactive protein
TMD: Time to metastatic disease

## References

[1] Chan DSM (2014). Body mass index and survival in women with breast cancer—systematic literature review and meta-analysis of 82 follow-up studies. Ann. Oncol..

[2] Gesta S, Kahn CR, Symonds ME (2017). White adipose tissue. Adipose Tissue Biology.

[3] Schaffler A, Scholmerich J (2010). Innate immunity and adipose tissue biology. Trends Immunol..

[4] Pond CM, Symonds ME (2017). The evolution of mammalian adipose tissues. Adipose Tissue Biology.

[5] Cinti S (2005). Adipocyte death defines macrophage localization and function in adipose tissue of obese mice and humans. J. Lipid Res..

[6] Giordano A (2013). Obese adipocytes show ultrastructural features of stressed cells and die of pyroptosis. J. Lipid Res..

[7] Quail DF, Dannenberg AJ (2019). The obese adipose tissue microenvironment in cancer development and progression. Nat. Rev. Endocrinol..

[8] Maliniak ML (2021). Crown-like structures in breast adipose tissue: Early evidence and current issues in breast cancer. Cancers.

[9] Iyengar NM (2015). Menopause is a determinant of breast adipose inflammation. Cancer Prev. Res..

[10] Iyengar NM (2016). Systemic correlates of white adipose tissue inflammation in early-stage breast cancer. Clin. Cancer Res..

[11] Bourlier V (2008). Remodeling phenotype of human subcutaneous adipose tissue macrophages. Circulation.

[12] Mullooly M (2017). Relationship between crown-like structures and sex-steroid hormones in breast adipose tissue and serum among postmenopausal breast cancer patients. Breast Cancer Res..

[13] Cha YJ, Kim ES, Koo JS (2018). Tumor-associated macrophages and crown-like structures in adipose tissue in breast cancer. Breast Cancer Res. Treat..

[14] Koru-Sengul T (2016). Breast cancers from black women exhibit higher numbers of immunosuppressive macrophages with proliferative activity and of crown-like structures associated with lower survival compared to non-black Latinas and Caucasians. Breast Cancer Res. Treat..

[15] Gonzalez-Angulo AM (2009). High risk of recurrence for patients with breast cancer who have human epidermal growth factor receptor 2-positive, node-negative tumors 1 cm or smaller. J. Clin. Oncol..

[16] Cronin KA, Harlan LC, Dodd KW, Abrams JS, Ballard-Barbash R (2010). Population-based estimate of the prevalence of HER-2 positive breast cancer tumors for early stage patients in the US. Cancer Investig..

[17] Mesa-Eguiagaray I (2020). Distinct temporal trends in breast cancer incidence from 1997 to 2016 by molecular subtypes: A population-based study of Scottish cancer registry data. Br. J. Cancer.

[18] Marty M (2005). Randomized phase II trial of the efficacy and safety of trastuzumab combined with docetaxel in patients with human epidermal growth factor receptor 2-positive metastatic breast cancer administered as first-line treatment: The M77001 study group. J. Clin. Oncol..

[19] Swain SM (2013). Pertuzumab, trastuzumab, and docetaxel for HER2-positive metastatic breast cancer (CLEOPATRA study): Overall survival results from a randomised, double-blind, placebo-controlled, phase 3 study. Lancet Oncol..

[20] Valabrega G, Montemurro F, Aglietta M (2007). Trastuzumab: Mechanism of action, resistance and future perspectives in HER2-overexpressing breast cancer. Ann. Oncol..

[21] Hudis CA (2007). Trastuzumab—Mechanism of action and use in clinical practice. N. Engl. J. Med..

[22] Shi Y (2015). Trastuzumab triggers phagocytic killing of high HER2 cancer cells in vitro and in vivo by interaction with Fcγ receptors on macrophages. J. Immunol..

[23] Clynes RA, Towers TL, Presta LG, Ravetch JV (2000). Inhibitory Fc receptors modulate in vivo cytotoxicity against tumor targets. Nat. Med..

[24] Honkanen TJ (2019). Prognostic and predictive role of tumour-associated macrophages in HER2 positive breast cancer. Sci. Rep..

[25] Wang Z (2019). Paradoxical effects of obesity on T cell function during tumor progression and PD-1 checkpoint blockade. Nat. Med..

[26] McQuade JL (2018). Association of body-mass index and outcomes in patients with metastatic melanoma treated with targeted therapy, immunotherapy, or chemotherapy: A retrospective, multicohort analysis. Lancet Oncol..

[27] Martel S (2018). Impact of body mass index on the clinical outcomes of patients with HER2-positive metastatic breast cancer. Breast.

[28] Krasniqi E (2020). Impact of BMI on HER2+ metastatic breast cancer patients treated with pertuzumab and/or trastuzumab emtansine. Real-world evidence. J. Cell Physiol..

[29] Di Cosimo S (2020). Effect of body mass index on response to neo-adjuvant therapy in HER2-positive breast cancer: An exploratory analysis of the NeoALTTO trial. Breast Cancer Res..

[30] Mazzarella L (2013). Obesity increases the incidence of distant metastases in oestrogen receptor-negative human epidermal growth factor receptor 2-positive breast cancer patients. Eur. J. Cancer.

[31] AbdulJabbar K (2020). Geospatial immune variability illuminates differential evolution of lung adenocarcinoma. Nat. Med..

[32] Keren L (2018). A structured tumor-immune microenvironment in triple negative breast cancer revealed by multiplexed ion beam imaging. Cell.

[33] McNamara KL (2021). Spatial proteomic characterization of HER2-positive breast tumors through neoadjuvant therapy predicts response. medRxiv.

[34] Tutt AL (2015). Development and characterization of monoclonal antibodies specific for mouse and human Fcγ receptors. J. Immunol..

[35] Benjamini Y, Hochberg Y (1995). Controlling the false discovery rate: A practical and powerful approach to multiple testing. J. R. Stat. Soc. Ser. B (Methodological).

[36] Penman AD, Johnson WD (2006). The changing shape of the body mass index distribution curve in the population: Implications for public health policy to reduce the prevalence of adult obesity. Prev. Chronic Dis..

[37] Russo L, Lumeng CN (2018). Properties and functions of adipose tissue macrophages in obesity. Immunology.

[38] Schaffler A, Scholmerich J, Salzberger B (2007). Adipose tissue as an immunological organ: Toll-like receptors, C1q/TNFs and CTRPs. Trends Immunol..

[39] Kratz M (2014). Metabolic dysfunction drives a mechanistically distinct proinflammatory phenotype in adipose tissue macrophages. Cell Metab..

[40] Xu X (2013). Obesity activates a program of lysosomal-dependent lipid metabolism in adipose tissue macrophages independently of classic activation. Cell Metab..

[41] Shi H (2006). TLR4 links innate immunity and fatty acid-induced insulin resistance. J. Clin. Investig..

[42] Zuany-Amorim C, Hastewell J, Walker C (2002). Toll-like receptors as potential therapeutic targets for multiple diseases. Nat. Rev. Drug Discov..

[43] Iyengar NM (2017). Metabolic obesity, adipose inflammation and elevated breast aromatase in women with normal body mass index. Cancer Prev. Res..

[44] Iyengar NM (2018). Adiposity, inflammation, and breast cancer pathogenesis in Asian women. Cancer Prev. Res..

[45] Maliniak ML (2020). Detection of crown-like structures in breast adipose tissue and clinical outcomes among African-American and White women with breast cancer. Breast Cancer Res..

[46] Lumeng CN, Bodzin JL, Saltiel AR (2007). Obesity induces a phenotypic switch in adipose tissue macrophage polarization. J. Clin. Investig..

[47] Spector N, Blackwell K (2009). Understanding the mechanisms behind trastuzumab therapy for human epidermal growth factor receptor 2-positive breast cancer. J. Clin. Oncol..

[48] Giordano C (2013). Leptin increases HER2 protein levels through a STAT3-mediated up-regulation of Hsp90 in breast cancer cells. Mol. Oncol..

[49] Griner SE, Wang KJ, Joshi JP, Nahta R (2013). Mechanisms of adipocytokine-mediated trastuzumab resistance in HER2-positive breast cancer cell lines. Curr. Pharmacogenomics Person. Med..

[50] Soma D (2008). Leptin augments proliferation of breast cancer cells via transactivation of HER2. J. Surg. Res..

