# Supplementary material for: Prognostic significance of crown-like structures to trastuzumab response in patients with primary invasive HER2 + breast carcinoma
Source: Sci Rep. 2022 May 24;12:7802. doi: 10.1038/s41598-022-11696-6 (PMC9130517; doi:10.1038/s41598-022-11696-6)
Supplement: Supplementary file 1 — Supplementary Information. [file 41598_2022_11696_MOESM1_ESM.pdf]

Prognostic significance of crown-like structures to trastuzumab response in patients with primary invasive HER2+ breast carcinoma

Charles N Birts[1,2,3,†,*], Constantinos Savva[1,4,†], Stéphanie A Laversin[1], Alicia Lefas[1,4], Jamie Krishnan[1,4], Aron Schapira[1,4], Margaret Ashton-Key[1,4,5], Max Crispin[2,3], Peter WM Johnson[4,6], Jeremy P Blaydes[3,4], Ellen Copson[4,6,7], Ramsey I Cutress[4,6,7,*], Stephen A Beers[1,4,*]

[1]Antibody and Vaccine Group, Centre for Cancer Immunology, School of Cancer Sciences, Faculty of Medicine, University of Southampton, Southampton, SO16 6YD, UK. [2]School of Biological Sciences, Faculty of Environmental and Life Sciences, University of Southampton, Southampton, SO17 1BJ, UK. [3]Institute for Life Sciences, University of Southampton, Southampton, SO17 1BJ, UK. [4]CRUK Southampton Centre, School of Cancer Sciences, Faculty of Medicine, University of Southampton, Southampton, SO16 6YD, UK. [5]Cellular Pathology, University Hospitals Southampton NHS Foundation Trust, Southampton, SO16 6YD, UK.[6]Southampton Experimental Cancer Medicine Centre, School of Cancer Sciences, Faculty of Medicine, University of Southampton, Southampton, SO16 6YD, UK. [7]NIHR Southampton Biomedical Research Centre, University of Southampton and University Hospital Southampton NHS Foundation Trust, Southampton, SO16 6YD, UK. †Equal contribution, *Corresponding authors.

Supplementary figures and tables

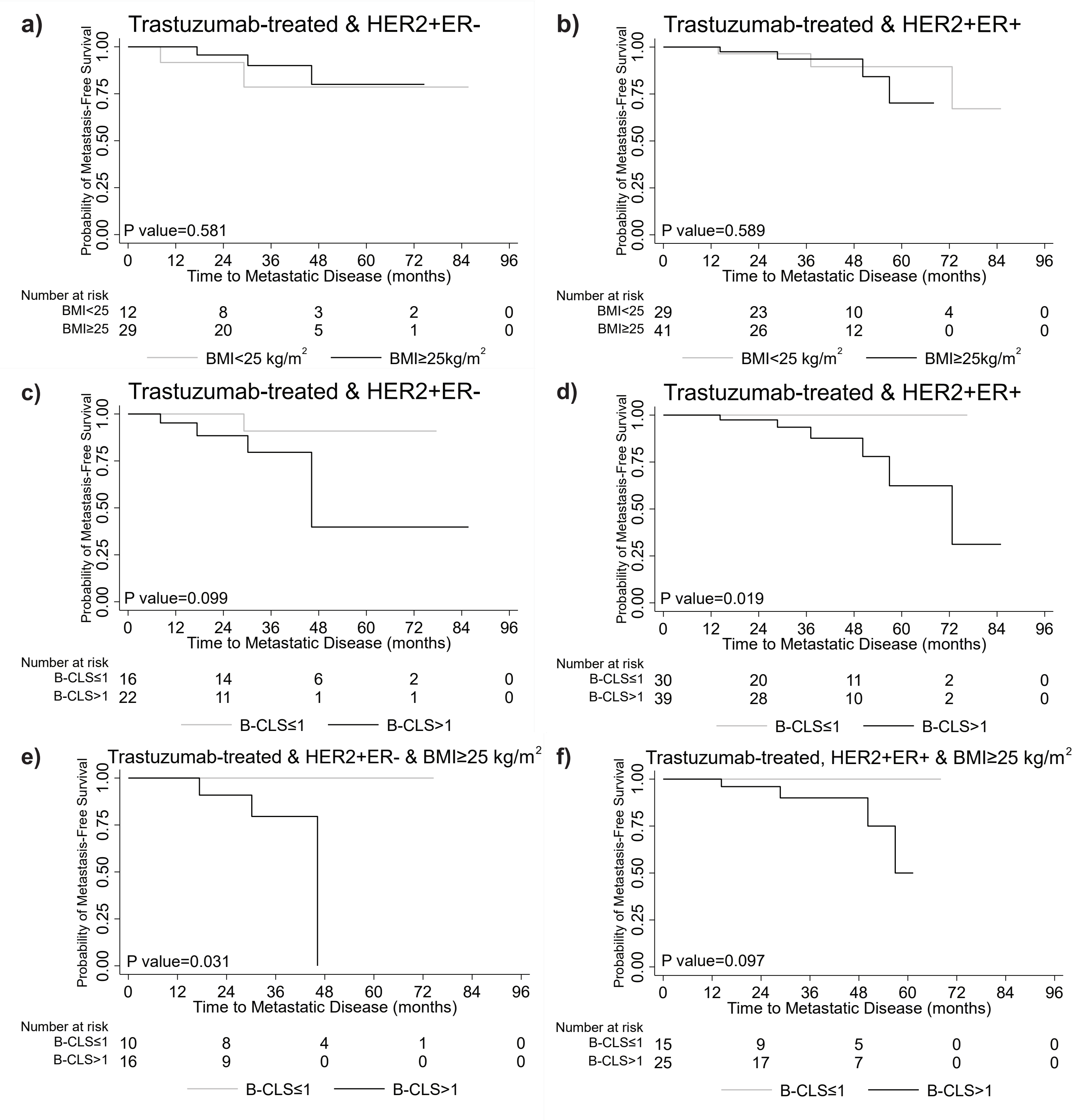

Supplementary Figure 1. Kaplan-Meier curves showing time to metastatic disease in trastuzumab-treated patients stratified by HER2 and ER profile. Comparison of patients with a-b) BMI<25 kg/m^2 versus BMI≥25 kg/m^2 c-d) B-CLS≤1 versus B-CLS>1 and e-f) B-CLS≤1 versus B-CLS>1 in patients with BMI ≥ 25 kg/m^2.

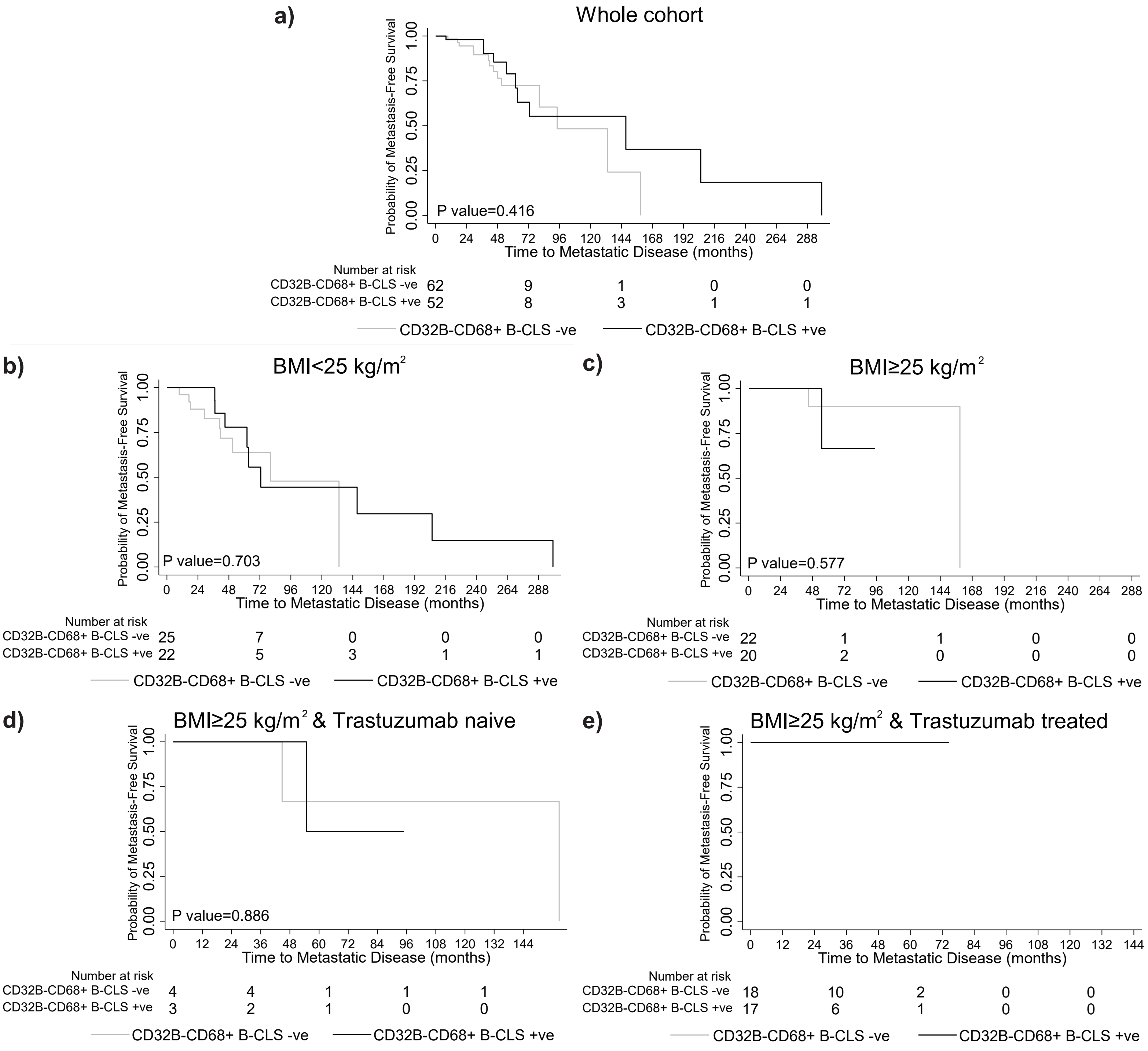

Supplementary Figure 2. Kaplan Meier curves showing time to metastatic disease in patients with CD32B- B-CLS. a) All patients in cohort. b-c) Patients stratified by BMI. d-e) Patients with BMI≥25 kg/m^2 stratified by trastuzumab treatment status.

**Supplementary table 1.** Baseline characteristics of the whole cohort of patients with primary HER2+ breast cancer (n=188).		
**Variable**	**N[a]**	**N (%)**
**CLS**	176	
D-CLS>1		30 (17.1)
D-CLS≤1		146 (82.9
B-CLS>1		97 (55.1)
B-CLS≤1		79 (44.9)
T-CLS>1		20 (11.4)
T-CLS≤1		156 (88.6)
Any-CLS>1		108 (61.4)
Any-CLS≤1		68 (38.6)
**Age (years)**	186	
<49		64 (34.4)
50-59		55 (29.6)
60-69		41 (22.0)
70-79		20 (10.8)
80-89		6 (3.2)
**BMI (kg/m^2)**	151	
<25		65 (43.1)
≥25		86 (57.0)
**T stage [b]**	187	
T1		82 (43.9)
T2		83 (44.4)
T3		21 (11.2)
T4		1 (0.5)
**Lymph node stage [b]**	186	
N0		77 (41.4)
N1		92 (49.5)
N2		13 (7.0)
N3		4 (2.2)
**Tumour Grade [c]**	186	
Grade 1		12 (6.5)
Grade 2		41 (22.0)
Grade 3		133 (71.5)
**Tumour type**	188	
Ductal		169 (89.9)
Lobular		3 (1.6)
Mixed Ductal/Lobular		8 (4.3)
Mixed other		4 (2.1)
Other		4 (2.1)
**ER status**	185	
Negative		63 (34.1)
Positive		122 (66.0)
**PR status**	70	
Negative		49 (70.0)
Positive		21 (30.0)
**Oncological treatment**		
Adjuvant chemotherapy	179	139 (77.7)
Post-op Radiotherapy	181	133 (73.5)
**Adjuvant Trastuzumab**	186	
Yes		117 (62.9)
No		69 (37.1)
**Haematological counts** (10^9/L) [d]	165	
White cells (≥ 9)		30(18.2)
Neutrophils (≥5.4)		49 (29.7)
Platelets (≥290)		49 (29.7)

Abbreviations: D-CLS, Distant adipose tissue CLS; B-CLS, CLS at the adipose-tumour border; T-CLS, intratumoural CLS; Any-CLS, CLS at any location; a, number of cases for which data was available; b, as per TNM Classification of Malignant Tumours (8th edition); c, as defined by Nottingham grading system; d Cut-off defined by ROC analysis.

**Supplementary table 2.** Missing data for clinical parameters by treatment status (n=186) †.			
**Variable**	**N[a]**	**Trastuzumab naive (n=69)**	**Adjuvant trastuzumab (n=117)**
D-CLS	12	3 (4.4)	9 (7.7)
B-CLS	12	3 (4.4)	9 (7.7)
T-CLS	12	3 (4.4)	9 (7.7)
Any-CLS	12	3 (4.4)	9 (7.7)
Age (years)	2	2 (2.9)	0 (0)
BMI (kg/m^2)	37	32 (46.4)	5 (4.3)
T stage [b]	1	1 (1.5)	0 (0)
Lymph node stage [b]	2	1 (1.5)	1 (0.9)
Tumour Grade [c]	2	2 (2.9)	0 (0)
Tumour type	4	4 (5.8)	0 (0)
ER status	3	3 (4.4)	0 (0)
PR status	118	44 (63.8)	73 (62.4)
Adjuvant chemotherapy	9	8 (11.6)	1 (0.9)
Post-op Radiotherapy	7	4 (5.8)	3 (2.6)
**Haematological counts** (10^9/L) [d]			
White cells	53	28 (40.6)	25 (21.4)
Neutrophils	23	19 (27.5)	4 (3.4)
Platelets	49	14 (20.3)	35 (29.9)

Abbreviations: †Trastuzumab treatment status was available for 186 patients; D-CLS, Distant adipose tissue CLS; B-CLS, CLS at the adipose-tumour border; T-CLS, intratumoural CLS; Any-CLS, CLS at any location; a, number of cases for which data was missing; b, as per TNM Classification of Malignant Tumours (8th edition); c, as defined by Nottingham grading system.

Supplementary table 3. Distant recurrences in trastuzumab-treated patients.

Follow-up (years)	Number of patients	Distant recurrence (n)	Lost (n)	Relapse Free Survival (%)	Standard Error (%)	[95% Conf. Int.]
CLS≤1						
0 to 1	47	0	5	1.0000	0.0000	. .
1 to 2	42	0	7	1.0000	0.0000	. .
2 to 3	35	1	10	0.9667	0.0328	0.7861 0.9952
3 to 4	24	0	6	0.9667	0.0328	0.7861 0.9952
4 to 5	18	0	9	0.9667	0.0328	0.7861 0.9952
5 to 6	9	0	5	0.9667	0.0328	0.7861 0.9952
6 to 7	4	0	4	0.9667	0.0328	0.7861 0.9952
CLS>1						
0 to 1	61	1	4	0.9831	0.0168	0.8857 0.9976
1 to 2	56	2	15	0.9425	0.0324	0.8315 0.9812
2 to 3	39	2	13	0.8845	0.0500	0.7388 0.9515
3 to 4	24	2	11	0.7889	0.0779	0.5849 0.9005
4 to 5	11	2	4	0.6136	0.1250	0.3308 0.8060
5 to 6	5	0	2	0.6136	0.1250	0.3308 0.8060
6 to 7	3	1	0	0.4091	0.1866	0.0880 0.7198
7 to 8	2	0	2	0.4091	0.1866	0.0880 0.7198

Supplementary table 4. Distant recurrences in trastuzumab-naïve patients.

Follow-up (years)	Number of patients	Distant recurrence (n)	Lost (n)	Relapse Free Survival (%)	Standard Error (%)	[95% Conf. Int.]
CLS≤1						
0 to 1	31	1	5	0.9649	0.0345	0.7760 0.9950
1 to 2	25	2	1	0.8861	0.0621	0.6864 0.9619
2 to 3	22	1	2	0.8439	0.0720	0.6346 0.9387
3 to 4	19	3	2	0.7033	0.0954	0.4728 0.8476
4 to 5	14	1	2	0.6492	0.1022	0.4137 0.8094
5 to 6	11	1	1	0.5874	0.1096	0.3471 0.7652
6 to 7	9	1	1	0.5183	0.1165	0.2767 0.7144
7 to 8	7	3	0	0.2961	0.1176	0.0996 0.5263
8 to 9	4	0	2	0.2961	0.1176	0.0996 0.5263
11 to 12	2	1	0	0.1481	0.1201	0.0124 0.4356
13 to 14	1	1	0	0.0000	.	. .
CLS>1						
0 to 1	34	1	3	0.9692	0.0303	0.8010 0.9956
1 to 2	30	1	3	0.9352	0.0444	0.7644 0.9834
2 to 3	26	1	4	0.8963	0.0571	0.7098 0.9656
3 to 4	21	2	5	0.7994	0.0824	0.5759 0.9131
4 to 5	14	2	2	0.6764	0.1061	0.4239 0.8368
5 to 6	10	1	0	0.6087	0.1150	0.3510 0.7903
7 to 8	9	1	2	0.5327	0.1233	0.2741 0.7360
8 to 9	6	0	1	0.5327	0.1233	0.2741 0.7360
10 to 11	5	2	0	0.3196	0.1382	0.0909 0.5812
12 to 13	3	1	0	0.2131	0.1267	0.0374 0.4831
17 to 18	2	1	0	0.1065	0.0984	0.0066 0.3688
24 to 25	1	1	0	0.0000	.	. .

Supplementary table 5. Baseline characteristics of 69 trastuzumab naive patients with primary HER2+ breast cancer by B-CLS status.

Variable n (%)	N[a]	Trastuzumab naive			
**B-CLS**	66	**B-CLS ≤1 (n=31)**	**B-CLS >1 (n=35)**	**P value [e]**	**P value [f]**
**Age (years)**	64				
<49		5 (16.7)	12 (35.3)	0.346	0.730
50-59		7 (23.3)	5 (14.7)		
60-69		10 (33.3)	6 (17.7)		
70-79		6 (20.0)	8 (23.5)		
80-89		2 (6.7)	3 (8.8)		
**BMI (kg/m^2)**	35				
<25		11 (58.8)	10 (55.6)	0.845	0.856
≥25		7 (41.2)	8 (44.4)		
**T stage [b]**	65				
T1		13 (41.9)	21 (61.7)	**0.026**	0.280
T2		16 (51.6)	7 (20.6)		
T3		2 (6.5)	6 (17.6)		
**Lymph node stage [b]**	65				
N0		14 (45.2)	14 (41.2)	0.806	0.856
N1		16 (51.6)	18 (52.9)		
N2		1 (3.2)	1 (2.9)		
N3		0 (0)	1 (2.9)		
**Tumour Grade [c]**	64				
Grade 1		4 (13.8)	5 (14.3)	0.784	0.856
Grade 2		7 (24.1)	6 (17.1)		
Grade 3		18 (62.1)	24 (68.6)		
**Tumour type**	66				
Ductal		23 (74.2)	32 (91.4)	0.154	0.539
Lobular		3 (9.7)	0 (0)		
Mixed Ductal/Lobular		2 (6.5)	2 (5.7)		
Other		3 (9.7)	1 (2.9)		
**ER status**	63				
Negative		9 (31.0)	9 (26.5)	0.689	0.856
Positive		20 (69.0)	25 (73.5)		
**PR status**	22				
Negative		10 (71.4)	6 (75.0)	0.856	0.856
Positive		4 (28.6)	2 (25.0)		
**Treatment**					
Adjuvant chemotherapy	58	12 (44.4)	11 (35.5)	0.487	0.852
Post-op Radiotherapy	62	22 (78.6)	22 (64.7)	0.321	0.730
**Haematological counts (**10^9/L**)** [d]	49				
White cells (≥ 9)		7 (30.4)	2 (7.7)	**0.040**	0.280
Neutrophils (≥5.4)		11 (47.8)	7 (26.9)	0.130	0.539
Platelets (≥290)		8 (34.8)	6 (23.1)	0.365	0.730

Abbreviations: B-CLS, CLS at the adipose-tumour border; a, number of cases for which data was available; b, as per TNM Classification of Malignant Tumours (8th edition); c, as defined by Nottingham grading system; d Cut-off defined by ROC analysis; e, unadjusted p-value, Pearson's chi-squared or Fisher's exact test for categorical and t-test for continuous variables. f, Benjamini-Hochberg multiple comparison test.

Supplementary table 6. Baseline characteristics of 117 trastuzumab-treated patients with primary HER2+ breast cancer by B-CLS status.

**Variable n (%)**	**N[a]**	**Trastuzumab-treated**			
**B-CLS**	108	**B-CLS ≤1 (n=47)**	**B-CLS >1 (n=61)**	**P value [e]**	**P value [f]**
**Age (years)**	108				
<49		19 (40.4)	20 (32.8)	0.734	0.850
50-59		16 (34.0)	24 (39.3)		
60-69		11 (23.4)	13 (21.3)		
70-79		1 (2.1)	3 (4.9)		
80-89		0 (0)	1 (1.6)		
**BMI (kg/m²)**	103				
<25		18 (41.9)	19 (31.7)	0.288	0.458
≥25		25 (58.1)	41 (68.3)		
**T stage [b]**	108				
T1		20 (42.6)	22 (36.1)	0.251	0.458
T2		25 (53.2)	29 (47.5)		
T3		2 (4.3)	9 (14.8)		
T4		0 (0)	1 (1.6)		
**Lymph node stage [b]**	107				
N0		17 (37.0)	25 (41.0)	**0.005**	0.075
N1		26 (56.5)	26 (42.6)		
N2		0 (0)	10 (16.4)		
N3		3 (6.5)	0 (0)		
**Tumour Grade [c]**	108				
Grade 1		2 (4.3)	1 (1.6)	0.325	0.458
Grade 2		8 (17.0)	17 (27.9)		
Grade 3		37 (78.7)	43 (70.5)		
**Tumour type**	108				
Ductal		43 (91.5)	57 (93.4)	0.336	0.458
Lobular		0 (0)	0 (0)		
Mixed Ductal/Lobular		1 (2.1)	3 (4.9)		
Other		3 (6.4)	1 (1.6)		
**ER status**	108				
Negative		16 (34.0)	22 (36.1)	0.827	0.886
Positive		31 (66.0)	39 (63.9)		
**PR status**	41				
Negative		10 (58.8)	18 (75.0)	0.273	0.458
Positive		7 (41.2)	6 (25.0)		
**Treatment**					
Adjuvant chemotherapy	107	46 (100.0)	59 (96.7)	0.215	0.215
Post-op Radiotherapy	106	36 (78.3)	43 (71.7)	0.587	0.734
Length of Trastuzumab, mean (SD) (months)	106	10.80 (2.4)	10.24 (2.8)	0.286	0.458
**Haematological counts** (10^9/L) [d]	105				
White cells (≥ 9)		5 (11.4)	16 (26.2)	0.060	0.420
Neutrophils (≥5.4)		9 (20.5)	20 (32.8)	0.163	0.458
Platelets (≥290)		9 (20.5)	22 (36.1)	0.084	0.420

Abbreviations: B-CLS, CLS at the adipose-tumour border; a, number of cases for which data was available; b, as per TNM Classification of Malignant Tumours (8th edition); c, as defined by Nottingham grading system; d Cut-off defined by ROC analysis; e, unadjusted p-value, Pearson's chi-squared or Fisher's exact test for categorical and t-test for continuous variables. f, Benjamini-Hochberg multiple comparison test.

Supplementary table 7. Univariate and multivariate analysis for time to metastatic disease of the trastuzumab-treated patients comparing multiple CD32B+ B-CLS +ve vs CD32B+ B-CLS -ve patients.

Variable	N[a]	Univariate HR (95%CI)	P-value	Multivariate HR (95%CI)	P-value
CD32B+ B-CLS>1 +ve versus CD32B+ B-CLS -ve[b]	99	5.83 (1.4-23.7)	**0.014**	4.9 (1.2-20.6)	**0.029**
BMI ≥25 versus <25 kg/m^2	111	1.1 (0.3-3.5)	0.914		
T3/4 versus T1/2 tumour stage [c]	116	2.8 (0.8-10.4)	0.123		
N2/3 versus N0/1 nodal stage [c]	115	3.8 (0.8-18.7)	0.097		
Tumour grade 3 versus grade 1/2 [d]	116	0.9 (0.2-3.2)	0.841		
HER2+ER- versus HER2+ER+	115	1.3 (0.4-4.2)	0.642		
White cell count (≥9 versus <9 10^9/L)	112	3.8 (1.2-12.6)	**0.028**	1.9 (0.4-8.0)	0.404
Neutrophils (≥5.4 versus <5.4 10^9/L)	112	2.8 (0.9-8.8)	0.072		
Platelets (≥290 versus <290 10^9/L)	112	2.7 (0.9-8.3)	0.094		

Abbreviations: B-CLS, CLS at the adipose-tumour border; a, number of cases for which data was available; b, CD32B+ B-CLS -ve, patients with CD32B- B-CLS, CD32B+ B-CLS≤1, no B-CLS or any CLS; c, as per TNM Classification of Malignant Tumours (8th edition); d, as defined by Nottingham grading system; Bold, statistically significant.
